# Clinical Research Informatics: Contributions from 2023

**DOI:** 10.1055/s-0044-1800733

**Published:** 2025-04-08

**Authors:** Xavier Tannier, Dipak Kalra

**Affiliations:** 1 Sorbonne University, Inserm, University Sorbonne Paris-Nord, University Paris 13, Sorbonne Paris Cité, INSERM UMR_S 1142, LIMICS, F-75006 Paris, France; 2 The University of Gent, Gent, Belgium

**Keywords:** International Medical Informatics Association Yearbook, Clinical Research Informatics, Biomedical Research, Clinical Trials as Topic, Observational studies as Topic, Real-world data, Health Data Quality, Phenotyping

## Abstract

**Objectives** : To summarize key contributions to current research in the field of Clinical Research Informatics (CRI) and to select the best papers published in 2023.

**Methods** : A bibliographic search using a combination of MeSH descriptors and free-text terms on CRI was performed using PubMed, followed by a double-blind review in order to select a list of candidate best papers to be then peer-reviewed by external reviewers. After peer-review ranking, a consensus meeting between the two section editors and the editorial team was organized to finally conclude on the selected three best papers.

**Results** : Among the 1,119 papers returned by the search, published in 2023, that were in the scope of the various areas of CRI, the full review process selected three best papers. The first best paper describes the process undertaken in Germany, under the national Medical Informatics Initiative, to define and validate a provenance metadata framework to enable the interpretation including quality assessment of health data reused for research. The authors of the second-best paper present a methodology for the generation of computable phenotypes and the covariates associated with success rates in e-phenotype validation. The third-best presents a review of published and accessible tools that enable the assessment of health data quality through an automated process. This year's survey paper marks the tenth anniversary of the CRI section of the Yearbook by reviewing the dominant themes within CRI over the past decade and the major milestone innovations within this field.

**Conclusions** : The literature relevant to CRI in 2023 has largely been populated by publications that assess and enhance the reusability of health data for clinical research, in particular data quality assessment and metadata management.

## 1. Introduction

Within the 2023 International Medical Informatics Association (IMIA) Yearbook, the goal of the Clinical Research Informatics (CRI) section is to provide an overview of research trends from 2023 publications that demonstrate the progress in multifaceted aspects of medical informatics supporting research and innovation in the healthcare domain. New methods, tools, and CRI systems have been developed in order to enable real-world evidence generation and optimize the life-cycle of clinical trials. Although this year's Yearbook focus should ideally be on equity, for this year we found only one publication in CRI that takes this inclusivity perspective. The selections made have therefore focused on maximising the reusability of health data within clinical research through the use of real world data, which is more inclusive than conventional clinical trial data.

## 2. About the paper Selection

A comprehensive review of articles published in 2023 and addressing a wide range of issues for CRI was conducted. The selection was performed by querying MEDLINE via PubMed (from NCBI, National Center for Biotechnology Information) with a set of predefined MeSH descriptors and free terms: Clinical research informatics, Biomedical research, Nursing research, Clinical research, Medical research, Pharmacovigilance, Patient selection, Phenotyping, Genotype-phenotype associations, Feasibility studies, Eligibility criteria, Feasibility criteria, Cohort selection, Patient recruitment, Clinical trial eligibility screening, Eligibility determination, Patient-trial matching, Protocol feasibility, Real world evidence, Data Collection, Epidemiologic research design, Clinical studies as Topic, Multicenter studies as Topic, and Evaluation studies as Topic. Papers addressing topics of other sections of the Yearbook, such as Translational Bioinformatics, were excluded based on the predefined exclusion of MeSH descriptors such as Genetic research, Gene ontology, Human genome project, Stem cell research, or Molecular epidemiology.

Bibliographic databases were searched twice, initially in November 2023 and then refreshed for late in the year publications on January 15th 2024, considering the electronic publication date. A set of 1,119 papers were found and screened for relevance to CRI, for reporting original research as opposed to opinion or educational papers, and their scientific quality was blindly rated as low, medium, high or out of scope by the two section editors based on papers' title and abstract. A total of 55 references classified as high to medium quality contributions to the field by at least one section editor were considered and classified into the following areas of the CRI domain in order of the number of matching papers (multiple classification choices were permitted): reuse of EHR data, Real-World Evidence generation, electronic phenotyping; data integration, semantic interoperability and data quality assessment; security, confidentiality and data privacy; data/text mining, Artificial Intelligence and Machine Learning, communicating study results; feasibility studies, patient recruitment, data management, CRI systems and improved use experiences; ethical, legal, social, policy issues and solutions, stakeholder participation; research networks, team science, Learning Healthcare System, Health Technology Assessment. A further review by both editors after full paper screening resulted in 11 candidate papers. In conformance with the IMIA Yearbook process, these 11 papers were peer-reviewed by the IMIA Yearbook editors and external reviewers. Each paper was reviewed by exactly 5 reviewers. Three papers were finally selected as best papers ( Table 1 ). A content summary of these best papers can be found in the appendix of this synopsis.

**Table 1.** Selection of best papers for the 2023 IMIA Yearbook of Medical Informatics for the section Clinical Research Informatics. The articles are listed in alphabetical order of the first author's surname.

Gierend K, Waltemath D, Ganslandt T, Siegel F. Traceable Research Data Sharing in a German vMedical Data Integration Center With FAIR (Findability, Accessibility, Interoperability, and Reusability)-Geared Provenance Implementation: Proof-of-Concept Study. JMIR Form Res 2023;7:e50027. doi: 10.2196/50027
Hamidi B, Flume P, Simpson K, Alekseyenko A. Not all phenotypes are created equal: covariates of success in e-phenotype specification. JAMIA 2023;30:213–221. doi: 10.1093/jamia/ocac157
Ozonze O, Scott PJ, Hopgood, AA. Automating Electronic Health Record Data Quality Assessment. J Med Syst 47, 23 (2023). doi : 10.1007/s10916-022-01892-2

## 3. Outlook

The 11 candidate best papers for 2023 illustrate recent efforts and trends in different CRI areas: using real world data to assess clinical trial eligibility, metadata regarding integrity, traceability, and quality within research data sets, extracting computable meaning from clinical narratives, the representation of computable phenotypes, data quality assessment and quality management, federated learning models for AI and an analysis of factors affecting inclusivity of representation in clinical trials. This year's survey paper marks the tenth anniversary of the CRI section of the Yearbook by reviewing the dominant themes within CRI over the past decade and the major milestone innovations within this field.

### 3.1. The Reuse of Real World Datasets for Clinical Research

There is an increasing adoption of the FAIR principles and FAIR metadata to describe data sets for the purposes of reuse such as research. However, conventional practice does not include the detailed provenance metadata that captures the transformations from original source data to a data set that is deemed suitable for research. This year's first ranking best paper has established and validated the traceability provenance information of these transformations, to increase the potential for users to trust the quality of the data sets they access [ 1 ]. This paper is summarised in the appendix to this section.

### 3.2. Clinical Research Applications of NLP

The successful reuse of health data depends upon its computable content, but there is still a substantial proportion of useful clinical information held in narratives. One of the shortlisted papers tackles this in reporting challenges and lessons learned by the NLP working group of the OHDSI (Observational Health Data Sciences and Informatics) consortium [ 2 ]. The authors have developed a standardised workflow for the ETL process using various NLP tools (e.g., cTAKES, MetaMap Lite, CLAMP) to process and integrate textual notes into the structured format of the OMOP CDM. This should significantly advance the utilisation of clinical textual data in observational studies by providing a robust method to integrate such data into a widely used data model. Another shortlisted paper also applied NLP to clinical narratives, adding an artificial intelligence processing step and a reference repository of active clinical trial eligibility criteria to alert clinicians of a patient's potential eligibility within their routinely used EHR system [ 3 ]. The aim of the authors was to make clinicians aware of a patient's eligibility in a context closer to their clinical practice and to reduce the burden of manual case note screening for potential trial subjects. Linking to the next heading on computable phenotypes is a paper that utilised the Human Phenome Ontology (HPO) to improve the semantic rigour and precision of NLP when searching for computable phenotypes in large corpora of clinical narratives within EHR systems [ 4 ]. This approach benefits from the pre-defined relationships between terms in the HPO to improve the accuracy of phenotype identification and similarity compared to existing approaches.

### 3.3. Computable Phenotypes

Precise clinical codes for diagnoses and finer grained sub-types of a diagnosis are often absent in EHR data. The diagnostic specificity needed for clinical research, the eligibility of patients or details needed for the study, often must be inferred from other data points. For comparability these inferences need to be standardised: the criteria for defining a computable phenotype. These criteria are historically designed by clinical domain experts, which is expensive and time consuming. One of this year's best papers sought to understand the factors that contribute to the successful creation of electronic phenotypes (e-phenotypes) and to identify covariates associated with success rates in e-phenotype validation [ 5 ]. They tested the accuracy of computable phenotype definitions made by non-clinical informatics experts and found better performance in domains such as infectious diseases, rheumatic conditions, neonatal issues, and cancers, and worse performance in mental health and dermatology. The findings therefore emphasise the importance of specialist support in phenotype design to ensure high-quality, reliable e-phenotype creation. This paper is summarised in the appendix to this section. Another paper on computable phenotype selection criteria (rules) has examined the lack of consistency across definitions [ 6 ]. The authors took 33 existing phenotype definitions and translated them into a standardised format to examine their sophistication. They found that most definitions are relatively simplistic, with limited use of compound criteria with Boolean or other operators.

### 3.4. Data Quality

The assessment of data quality has proved year on year to be an increasing topic in the CRI literature, with strong publications as evidenced by the inclusion of this topic in this chapter in recent years. One of the three best papers is a review of the tools available to formally assess health data quality through automated methods, that are generic and scalable to adopt, as opposed to being tied to particular data architecture or specific to a disease area [ 7 ]. This paper is summarised in the appendix to this section. Another paper, shortlisted, focused on a method for conducting distributed data quality assessments i.e., without requiring the transfer of patient level data, as a framework for interoperable and privacy-preserving assessments [ 8 ]. The authors composed 25 measurable parameters covering four commonly used data quality dimensions: completeness, plausibility, uniqueness, conformance. This was validated using the Personal Health Train architecture across multiple data sources in a rare disease context. This is a useful proof of concept because rare disease research frequently needs to utilise data from multiple sources in order to reach sufficient patient numbers. Also in the set of candidate best papers is a contribution on data quality across the ETL pipeline from source EHRs to clinical datasets ready for research use [ 9 ]. Their quality control pipeline starts with a classical dimensional analysis of data quality in the source EHR before data is mapped to a common data model such as OMOP. The second layer examined the imported data for unexpected outliers, and the third examines the data as clusters during execution of test research queries.

### 3.5. Federated Machine Learning

The federated querying of a network of EHR systems has been progressively adopted over the past decade as a way of reducing the volumes of patient level data that have to be copied from healthcare providers into research centres. AI machine learning (ML) has proved the challenge to this model as the intensity of the analysis required, especially during model development, has proved too great up to now to perform in a federated manner, therefore being the exceptional use case that still required data centralisation. The CRI field is therefore very active in pursuing scalable and secure approaches to federated machine learning. One of the shortlisted papers this year reports on a novel privacy-preserving architecture for this, in which a convolutional (multi-layered) neural network is deployed at each healthcare site, enabling the most intensive processing to occur locally and with the results of processing (i.e., local ML models) stored locally [ 10 ]. A further privacy preserving feature is the use of Blockchain smart contracts, enabling patients to exercise control over this local processing. The centralised ML model is trained only on distributed access to the processed data at each site, through a Blockchain network. The authors have validated this architecture in a pilot using data on patients with Covid-19, with good performance, but recognise that further work is required to establish scalability, especially for image progressing.

### 3.6. Inclusivity within Clinical Trials

A thought-provoking paper that aligns with the over-arching theme of the 2024 Yearbook examines the factors that influence the observed lack of diversity in clinical research participation [ 11 ]. The authors discuss the barriers affecting the participation of minority or under-served populations, which include ethnicity, socio-economic factors and a mistrust of healthcare systems. The authors propose practical measures that clinical trial designers and clinical investigators could adopt to correct for this under-representation. The authors stress the importance of proactively looking for recruitment bias within the data, widening trial eligibility criteria and extending the range of clinical trial sites into communities that might be challenged with travelling to well-established (tertiary medical) centres. They also emphasise the importance of engaging patient communities in the design of clinical trial protocols and of educating communities about the importance and practicalities of trial participation.

### 3.7. The Last Ten Years of Clinical Research Informatics

Since this issue of the Yearbook marks the tenth year of the Clinical Research Informatics chapter, we asked two CRI experts to perform a ten-year retrospective review of the literature in this field. The two authors each have experience of conducting seminal paper reviews, Christel Daniel as the former editor of this Yearbook chapter, and Peter Embi who has for many years undertaken the AMIA year in review. The authors combined these two review processes to identify and screen 1,500 papers to shortlist 200 that have made milestone contributions to CRI. Their paper presents the dominant themes within our field over this ten-year period, highlighting the most influential works per theme. They also present a deeper dive look at the newer themes emerging over the past two years, in particular translational research and the better integration of care and research.
